# Jujuboside B Inhibits the Proliferation of Breast Cancer Cell Lines by Inducing Apoptosis and Autophagy

**DOI:** 10.3389/fphar.2021.668887

**Published:** 2021-09-24

**Authors:** Lin Guo, Yupei Liang, Shiwen Wang, Lihui Li, Lili Cai, Yongqing Heng, Jing Yang, Xing Jin, Junqian Zhang, Shuying Yuan, Tong Xu, Lijun Jia

**Affiliations:** ^1^ Cancer Institute, Fudan University Shanghai Cancer Center, Fudan University, Shanghai, China; ^2^ Department of Immunology, School of Basic Medical Sciences, Fudan University, Shanghai, China; ^3^ Cancer Institute, Longhua Hospital, Shanghai University of Traditional Chinese Medicine, Shanghai, China; ^4^ Department of Laboratory Medicine, Huadong Hospital Affiliated to Fudan University, Shanghai, China

**Keywords:** jujuboside B, breast cancer, apoptosis, autophagy, NOXA, AMPK

## Abstract

Jujuboside B (JB) is one of the main biologically active ingredients extracted from Zizyphi Spinosi Semen (ZSS), a widely used traditional Chinese medicine for treating insomnia and anxiety. Breast cancer is the most common cancer and the second leading cause of cancer-related death in women worldwide. The purpose of this study was to examine whether JB could prevent breast cancer and its underlying mechanism. First, we reported that JB induced apoptosis and autophagy in MDA-MB-231 and MCF-7 human breast cancer cell lines. Further mechanistic studies have revealed that JB-induced apoptosis was mediated by NOXA in both two cell lines. Moreover, the AMPK signaling pathway plays an important role in JB-induced autophagy in MCF-7. To confirm the anti-breast cancer effect of JB, the interaction of JB-induced apoptosis and autophagy was investigated by both pharmacological and genetic approaches. Results indicated that autophagy played a pro-survival role in attenuating apoptosis. Further *in vivo* study showed that JB significantly suppressed the growth of MDA-MB-231 and MCF-7 xenografts. In conclusion, our findings indicate that JB exerts its anti-breast cancer effect in association with the induction of apoptosis and autophagy.

## Introduction

In recent years, a variety of active ingredients, such as resveratrol, chamaejasmenin B, ginsenoside, and angelica polysaccharide (Zhou et al., 2015; Kong et al., 2016; Li et al., 2016; Tai et al., 2016; Wang et al., 2016; Elshaer et al., 2018; Luo et al., 2019), extracted from traditional Chinese herbs, were confirmed to have anti-cancer activity (You et al., 2013). These natural compounds exert anti-cancer effects by affecting multiple mechanisms, including inducing apoptosis, promoting necrosis, regulating autophagy, arresting cell cycle, balancing immunity, inhibiting metastasis, and enhancing chemotherapy *in vitro* and *in vivo* (Green and Llambi, 2015; D’Arcy, 2019; Luo et al., 2019).

Zizyphi Spinosi Semen (ZSS) is the seed of traditional Chinese medicine *Zizyphus jujuba* var. *spinosa,* which is widely used to treat insomnia and anxiety (Jiang et al., 2007; Zhang et al., 2008; Liu et al., 2018). Besides, its various pharmacological properties, such as a beneficial effect on cognition, anti-inflammatory activity, anti-oxidation activity, and anti-aging and anti-tumor activity, were also validated *in vitro* and *in vivo* (He et al., 2020). Many compounds have been identified in ZSS, including terpenoids, alkaloids, flavonoids, fatty acids, volatile oils, and polysaccharides (He et al., 2020). Among these components, jujuboside B (JB) has been reported to be a main active component exerting the sedative-hypnotic effect (Jiang et al., 2007; Zhang et al., 2008; Liu et al., 2018). Recently, JB was also found to have ant-itumor activity. Studies have shown that JB exhibited anti-leukemic activity by inducing necroptosis *via* activation of RIPK1/RIPK3/MLKL pathway and triggering apoptosis (Jia et al., 2020). JB inhibited the proliferation of AGS and HCT116 cell lines *in vitro* and *in vivo* through promoting apoptosis mediated by p38/JNK (Xu et al., 2014). However, the anti-tumor potential and mechanism of JB remain largely unknown.

Breast cancer remains a worldwide public health dilemma and is currently the most common tumor in women globally (DeSantis et al., 2019). It accounts for approximately 30% of all female malignancies worldwide and 15% of cancer-related deaths (DeSantis et al., 2019). Despite advances in prevention and therapeutics over recent decades, the morbidity of breast cancer still continues to increase, especially in developed countries. Considering the adverse reactions that frequently occur during/after chemotherapy and radiotherapy, the physical condition and life quality of breast cancer patients are usually very poor. It is reported that the majority of breast cancer patients have emotional disorders, such as insomnia, anxiety, depression, and even suicidal tendency (Kwak et al., 2020). To alleviate the adverse effects, traditional Chinese herbs, such as immunity-enhancing and anti-depression herbs, are frequently used as adjuvant therapies (Qi et al., 2015; Lee et al., 2020; Wang et al., 2020). Increasing evidence has shown that traditional Chinese herbs and their active ingredients have potential therapeutic effects on breast cancer (You et al., 2013; Qi et al., 2015; Lee et al., 2020; Wang et al., 2020). These studies have led to the hypothesis that JB could attenuate the progression of breast cancer.

To test this hypothesis, we investigated the anti-breast cancer potential of jujuboside B *in vitro* and *in vivo* and further studied its potential mechanism. To the best of our knowledge, this is the first study to investigate the anti-breast cancer effect and mechanism of jujuboside B.

## Materials and Methods

### Reagents

Jujuboside B was purchased from Sigma and dissolved in dimethyl sulfoxide (DMSO) to the concentration of 100 mM stock solution kept at −20°C. The primary antibodies to cleaved PARP, total PARP, LC3, p62, NOXA, beclin1, ATG7, AMPK, and phospho-AMPK were all purchased from Cell Signaling Technology (United States). The primary antibodies to *β*-actin and GAPDH were purchased from Santa Cruz Biotechnology (CA, United States). Antibodies to cleaved caspase-3, total caspase-3, and the secondary HRP-conjugated anti-rabbit and anti-mouse antibodies were purchased from HUABIO. BCA protein assay kit and protein page ruler were purchased from Thermo Fisher. The knockdown siRNA for NOXA, Beclin1, ATG7, AMPK, and control siRNA were purchased from GenePharma (Shanghai, China). The autophagy inhibitor chloroquine diphosphate (CQ) was purchased from Selleck.

### Cell Lines and Cell Culture

Human breast cancer cells MDA-MB-231 and MCF-7 were purchased from American Type Culture Collection (ATCC, Rockville, MD, United States) and cultured in Dulbecco’s Modified Eagle’s Medium (DMEM, GIBCO) supplemented with 10% fetal bovine serum (FBS, GIBCO) and 1% penicillin-streptomycin solution in a 5% CO_2_ incubator at 37°C.

### Cell Viability Assay

We chose an arithmetic sequence of 20, 40, 60, 80, and 100 µM to determine the antiproliferative activity and calculate IC50. MDA-MB-231 and MCF-7 cells were seeded into 96-well plates (3,000 cells per well) and treated with 0.1% DMSO or indicated concentrations of jujuboside B. After treatment for 72 h, cell viability was determined using ATPlite Luminescence Assay kit (PerkinElmer, Norwalk, CT, United States) according to the manufacturer’s instructions.

### Cell Clonogenic Assay

MDA-MB-231 and MCF-7 cells were seeded into 6-well plates (500 cells per well) in triplicate and treated with 0.1% DMSO or indicated concentrations of jujuboside B. After treatment for 10–14 days, the clones were fixed with 4% paraformaldehyde and stained with 0.05% crystal violet. After being washed, clones were counted under an inverted microscope. Representative results of three independent experiments with similar trends were presented.

### Cell Migration

It is a generally acknowledged fact that human breast cancer cell line MCF-7 is less flexible, non-metastatic, epithelium-like, and ER-positive cells. In comparison, MDA-MB-231 is more flexible, metastatic, aggressive, and mesenchymal-like breast cells with ER/PR/HER2 negative (Hu et al., 2019). Thus, cell migration assay was not applicable to MCF-7. A comparative migration assay was conducted in MDA-MB-231 cells using a 24-well transwell plate of 6.5 mm diameter with polycarbonate membrane filters containing 8 μm pores (Corning, NY, United States). 5*10^5^ cells suspended in 100 μL free DMEM containing DMSO or indicated concentrations of JB were seeded onto the upper room of the chamber. Meanwhile, 500 μL DMEM with 20% FBS was added to the lower wells of the chambers. Then, the transwell plate was incubated for 16 h in a 5% CO_2_ incubator at 37°C. The non-migrated cells were erased from the upper side of the membranes using cotton swabs. Cells that had gone through the membrane were fixed in 4% formaldehyde for 20 min and stained with 0.1% crystal violet solution for 30 min. The lower side of the membranes was photographed.

### Flow Cytometry

MDA-MB-231 and MCF-7 cells were seeded onto 6 cm dish (4*10^5^ per dish) in triplicate overnight and then treated with 0.1% DMSO or indicated concentrations of JB. After treatment for 48 h, cells were harvested by trypsin, washed by PBS, suspended in 300 μL Annexin V binding buffer, and stained with Annexin V-FITC/PI Apoptosis Kit (BD Biosciences, San Diego, United States) for 30 min at room temperature on ice in the dark. Then, samples were analyzed by flow cytometer (Beckman Coulter). Annexin V (+)/PI (−) cells were characterized as early apoptotic and Annexin V (+)/PI (+) as late apoptotic.

### siRNA Interference Silencing

MDA-MB-231 and MCF-7 cells were seeded onto 6 cm dish (4*10^5^ per dish) overnight in the incubator. The next day, cells were transiently transfected with siNOXA, siBeclin1, siATG7, siAMPK, or siNC using Lipofectamine™ RNAiMAX Transfection Reagent (Invitrogen, Carlsbad, CA, United States) according to the manufacturer’s instruction. Briefly, siRNA and RNAiMAX were incubated in Opti-MEM (Invitrogen) separately for 5 min at room temperature and mixed for 20 min, and then the mixture and serum-free medium were supplemented to the cells (final concentration of siRNA is 20 nM). After transfection for 6–8 h, the medium was changed back to normal medium containing FBS. All siRNAs were synthesized by GenePharma (Shanghai, China). The sequences of siRNAs were as follows:

siNOXA-1: 5ʹ-GUA​AUU​AUU​GAC​ACA​UUU​C-3ʹ
siNOXA-2: 5-GGU​GCA​CGU​UUC​AUC​AAU​UUG-3′
siBeclin1-1: 5ʹ-CAG​TTT​GGC​ACA​ATC​AAT​A-3ʹ
siBeclin1-2: 5ʹ-GGA​GGA​AGA​GAC​UAA​CUC​A-3ʹ
siATG7-1: 5ʹ-GGA​GUC​ACA​GCU​CUU​CCU​U-3ʹ
siATG7-2: 5ʹ-GAG​AUA​UGG​GAA​UCC​AUA​A-3ʹ
siAMPK-1: 5′-AGU​GAA​GGU​UGG​CAA​ACA​UTT-3ʹ
siAMPK-2: 5′-GGA​AGG​UAG​UGA​AUG​CAU​ATT-3ʹ
siNC: 5′-UUC​UCC​GAA​CGU​GUC​ACG​UTT-3ʹ

### Western Blot

After treatment with DMSO or indicated concentrations of JB for the appointed time, cells were harvested and rinsed twice with ice-cold PBS and lysed in RIPA lysis buffer containing PMSF and protease inhibitor cocktail (Beyotime, China). The supernatant liquid was collected after centrifugation at 12,000 × g at 4°C for 15 min and the protein concentration was determined by the BCA protein assay kit. After the addition of 5 × loading buffer, cell lysates were boiled at 95°C for 10 min. Lysates containing equal amounts of protein were loaded onto sulfate-polyacrylamide gels (SDS-PAGE) and transferred to PVDF membranes. The membranes were blocked with 5% slim milk in Tris-Buffered Saline with 0.1% Tween (TBST) at room temperature for 2 h and then incubated with primary antibodies at 4°C overnight followed by the corresponding HRP-conjugated secondary antibody at room temperature for 2 h. Blots signals were visualized by enhanced chemiluminescent (ECL) substrate and analyzed with *β*-actin or GAPDH used as the loading control. The primary antibodies included antibodies against cleaved PARP (C-PARP), total PARP (T-PARP), cleaved caspase-3 (C-cas3), total caspase-3 (T-cas3), NOXA, LC3, p62, AMPK, phospho-AMPK (P-AMPK), Beclin1, and ATG7.

### *In Vivo* Tumor Xenograft Model

A subcutaneous tumor model of breast cancer was established using both MCF-7 and MDA-MB-231 cell lines. Female 5-6-week-old athymic balb/c nude mice provided by Charles River Laboratories were housed in a specific pathogen-free environment and received food and water *ad libitum*. The SPF room is under a temperature of 22 ± 2°C with a 12 h light/12 h dark cycle and relative humidity of 40–60%. After the mice had been in quarantine for 1 week, 2*10^6^ MCF-7 cells suspended in a 100 μL mixture of PBS and matrigel (PBS: matrigel = 1:4) or 2*10^6^ MDA-MB-231 cells suspended in 100 μL PBS were injected subcutaneously into the right flank of each mouse. When the tumor volume reached 60 mm^3^, the mice were randomly allocated into two groups and treated intraperitoneally with vehicle (10% *β*-cyclodextrin) or JB 20 mg/kg every day. Mice weight and tumor volumes were recorded. At the end of the experiment, mice were euthanized and tumors were harvested, photographed, and weighed. All procedures of animal experiments were conducted in compliance with standard ethical guidelines and with the approval (202105007Z) of the Institutional Animal Care and Use Committee of Fudan University. According to its guidelines and suggestions, the experiment was finished before tumors grew to the permitted maximum length/width of no more than 20 mm. The MCF-7 tumors grew rapidly under the action of matrigel containing a number of growth factors and were harvested on D17. The MDA-MB-231 tumor model was established by 2*10^6^ cells suspended in PBS, and the tumors were harvested on D28. Tumor volume was calculated by vernier caliper using the following formula: tumor volume = length×width^2^/2.

### Statistical Analysis

All data were presented as mean ± SEM and the statistical difference between groups was assessed using GraphPad Prism 7 software (GraphPad Software, Inc., San Diego, CA, United States). Student’s *t*-test was used for the comparison of parameters between two groups. A *p*-value of *p* < 0.05 was considered to be significant; ns represents not significant. For all tests, three levels of difference significance (*p < 0.05, **p < 0.01, ***p < 0.001) were applied.

## Results

### Jujuboside B Inhibits the Proliferation and Migration of Human Breast Cancer Cells

**FIGURE 1:**
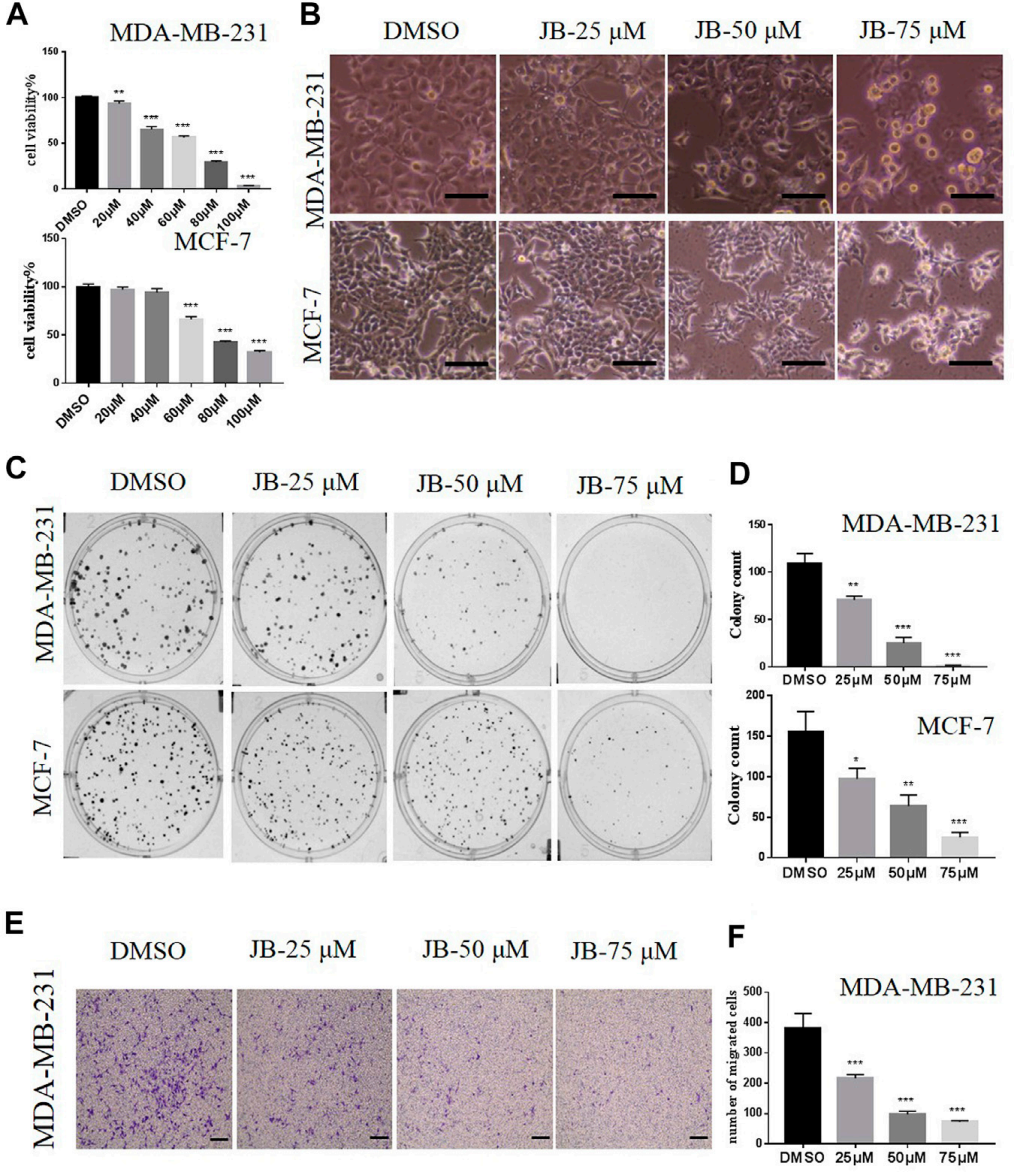
JB inhibits the proliferation of MDA-MB-231 and MCF-7 cells and inhibits the migration potential of MDA-MB-231 in a transwell assay. Cell viabilities were determined by ATPlite assay. The relative viability was calculated by the light unit vs. DMSO **(A)**. Cells were treated with DMSO or indicated concentrations of JB for 72 h followed by photographing, scale bar = 50 μm **(B)**; cells were treated with the indicated concentrations of JB for 10–14 days and representative images of clone formation assay were shown **(C)**; the values of colony number were analyzed and presented by mean ± SEM **(D)**. As described in the methods, a transwell assay was conducted in MDA-MB-231 cells; the lower side of the chamber membranes was photographed. scale bar = 100 μm **(E)**. The purple color stands for cells that have gone through the membrane. The numbers of migrated cells were gained from three random microscope fields in each chamber and were analyzed by mean ± SEM **(F)**. *p < 0.05 vs. DMSO group; **p < 0.01 vs. DMSO group; ***p < 0.001 vs. DMSO group, *n* = 3.

To verify our hypothesis that JB potentially has anti-breast cancer activity, we first evaluated the effect of JB on the proliferation of human breast cancer cell MDA-MB-231 and MCF-7. As shown in Figure 1A, cell viability assay by ATPlite showed that JB significantly inhibited the proliferation of MDA-MB-231 and MCF-7 in a dose-dependent manner. According to the cell viability results, the IC50 value for MDA-MB-231 is 54.38 and 74.94 μM for MCF-7. On overall consideration, we chose three concentrations of 25, 50, and 75 μM, ranging from lower than IC50 to higher than IC50, to conduct the following tests. Both MDA-MB-231 and MCF-7 cells phenotype distinctly changed, appeared round, and shrank after treatment by different concentrations of JB (Figure 1B). Clone formation assays were applied to detect the anti-clonogenic survival activity of JB. Results showed that JB obviously decreased the clone formation rate of both two cell lines (Figures 1C,D). It is well known that MDA-MB-231 is highly metastatic and prone to metastasis, while MCF-7 has little metastatic potential (Hu et al., 2019). Thus, we detected the anti-metastatic potential of JB in MDA-MB-231 cells. As shown in Figures 1E,F, JB inhibited the migration of MDA-MB-231 remarkably in a dose-dependent pattern.

### Jujuboside B Induces Cell Apoptosis in MDA-MB-231 and MCF-7 Cell Lines

**FIGURE 2:**
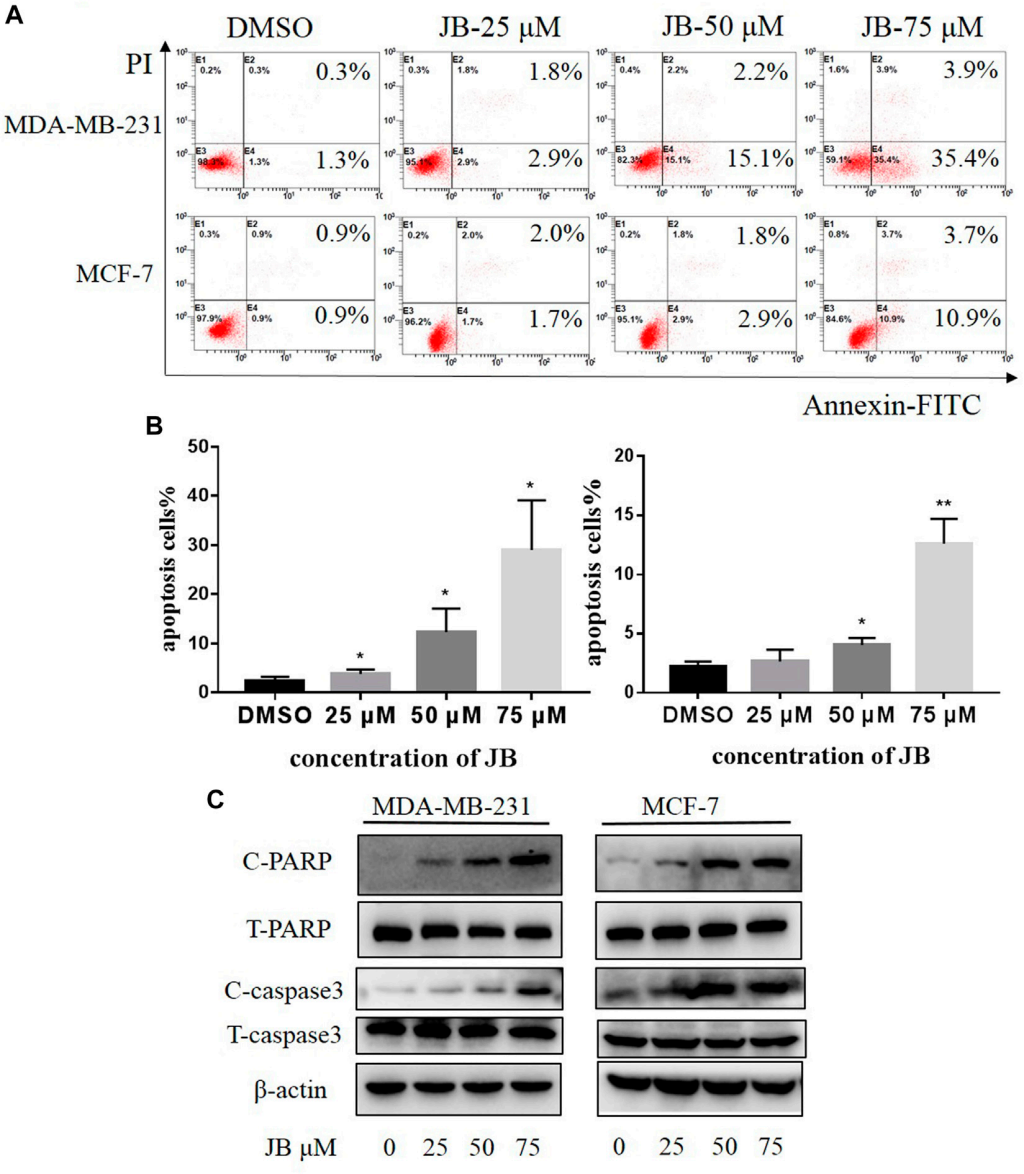
JB induces apoptosis in MDA-MB-231 and MCF-7 cell lines. Cells were treated with DMSO or indicated concentrations of JB for 48 h. Flow cytometry was performed to detect the apoptotic cell population percentage **(A)**. Statistical analysis of the relative apoptotic percentage vs. DMSO was presented by mean ± SEM **(B)**. Cleaved PARP, total PARP, cleaved caspase-3, and total caspase-3 were detected by western blot **(C)**. *p < 0.05 vs. DMSO group, **p < 0.01 vs. DMSO group, ***p < 0.001 vs. DMSO group, *n* = 3.

To explore whether JB affects cell apoptosis, MDA-MB-231 and MCF-7 were subjected to AnnexinV and PI staining. As depicted in Figures 2A,B, JB treatment resulted in a remarkable increase of the apoptotic cell population. To further confirm the effect of JB on cell apoptosis, we detected the expression of two classical molecular markers of apoptosis (cleaved PARP and cleaved caspase 3) in these two cell lines. Consistently, western blot revealed that JB significantly increased the expression of cleaved PARP and cleaved caspase-3 (Figure 2C). These results suggested that JB could inhibit the growth of MDA-MB-231 and MCF-7 by inducing apoptosis.

### Jujuboside B-Induced Apoptosis is Mediated by Pro-Apoptotic Protein NOXA

**FIGURE 3:**
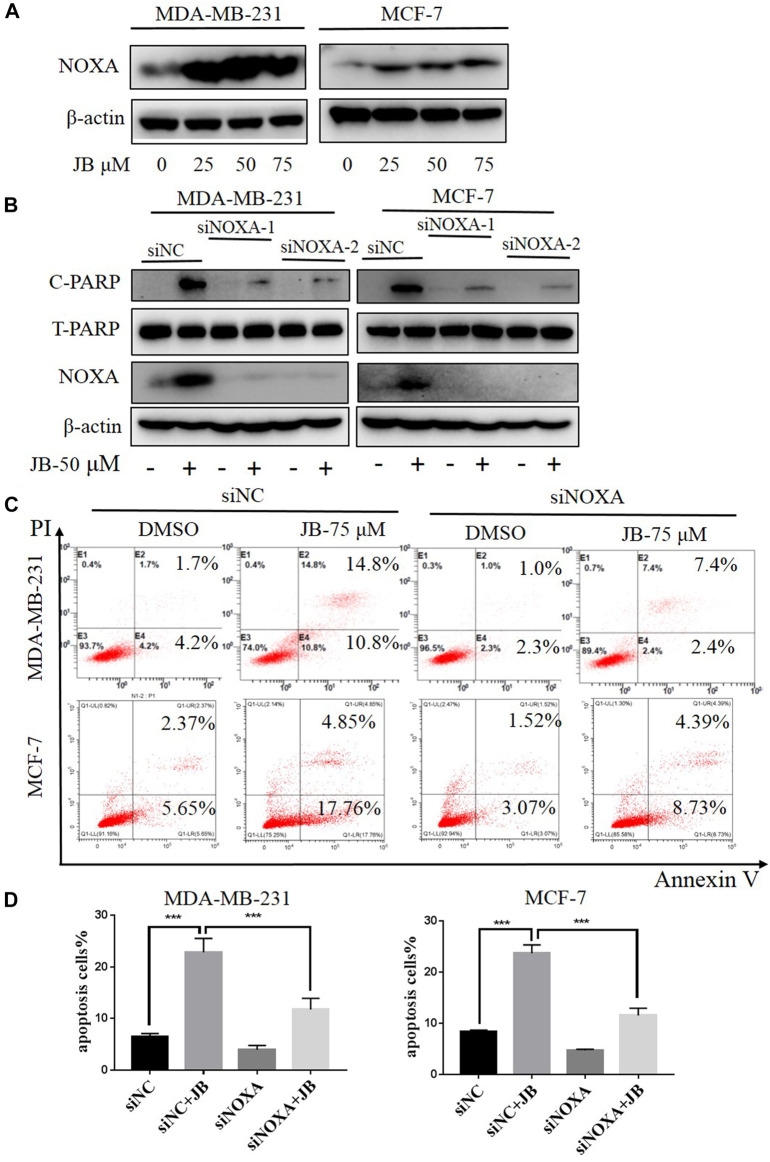
JB-induced apoptosis was mediated by NOXA. Cells were treated with DMSO or indicated concentrations of JB for 48 h and harvested for cell lysis preparation. The expression level of NOXA was detected by western blot **(A)**. Transfection of siRNA was conducted to knock down the expression of NOXA followed by treatment of JB for 48 h. The expression of NOXA, C-PARP, and T-PARP was detected by western blot **(B)**. Flow cytometry was performed to detect apoptosis **(C)**. Statistical analysis of the relative apoptotic percentages were presented by mean ± SEM **(D)**. *p < 0.05; **p < 0.01; ***p < 0.001, *n* = 3.

NOXA is a pro-apoptotic subset of the Bcl-2 family proteins. Previous studies have found that NOXA is a key factor that interacts with a variety of proteins during the process of apoptosis. Besides, NOXA plays an important role in the pathogenesis and treatment of a variety of cancers, especially in the endogenous apoptotic pathway (O’Prey et al., 2014; Morsi et al., 2018; Liang et al., 2019). To detect the effect of JB on NOXA, western blot was applied to analyze the expression of NOXA following treatment with indicated concentrations of JB. Results revealed that NOXA expression was remarkably elevated by JB in a dose-dependent pattern (Figure 3A), suggesting that JB-induced apoptosis may be associated with NOXA. To confirm it, we knocked down NOXA using two siRNA sequences and then detected the apoptosis level characterized by cleaved PARP. As shown in Figure 3B, NOXA was effectually downregulated by siNOXA-1 and siNOXA-2 transfection. As a result, the expression of cleaved PARP in both cell lines was reduced. In addition, flow cytometry confirmed that JB-induced apoptosis was dramatically attenuated after NOXA knockdown (Figures 3C,D). These results indicated that JB-induced apoptosis was mediated by NOXA.

### Jujuboside B Induces Autophagy in MDA-MB-231 and MCF-7 Cell Lines

**FIGURE 4:**
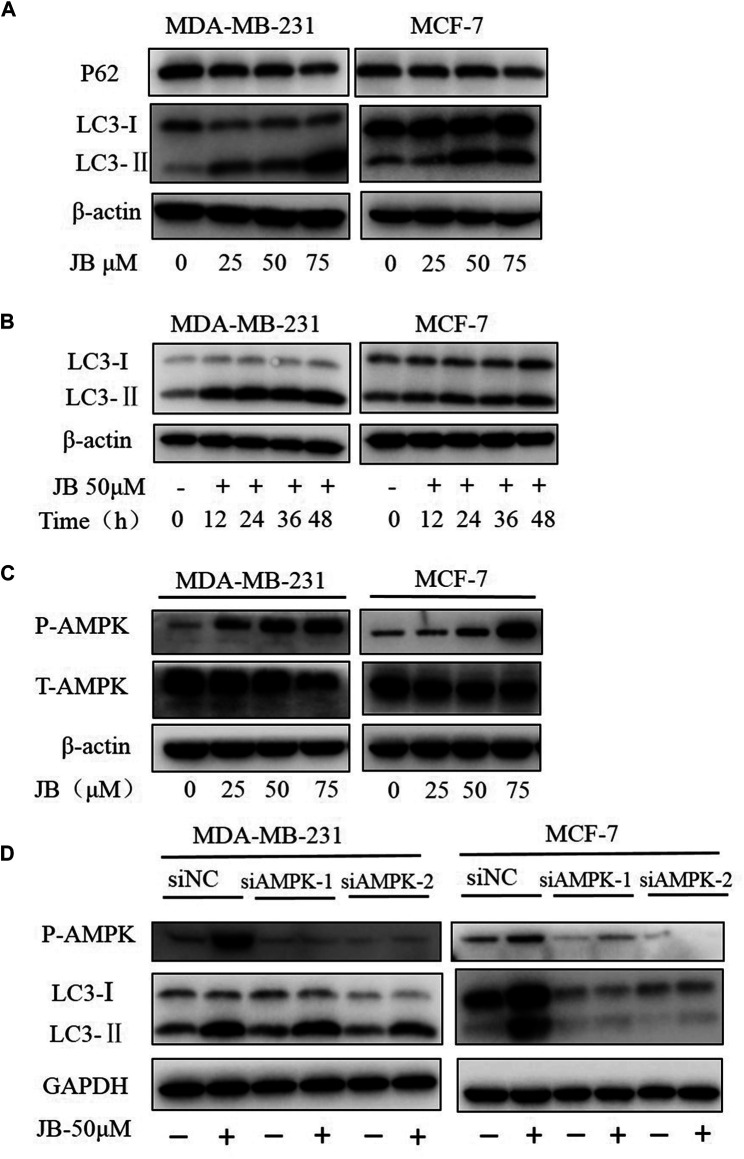
JB induces autophagy in MDA-MB-231 and MCF-7 cell lines. Cells were treated with DMSO or indicated concentrations of JB for 48 h and harvested for cell lysis preparation. The expressions of LC3-I, LC3-II, and p62 were detected by western blot **(A)**. Cells were treated with JB-50 μM in a time course of 0, 12, 24, 36, and 48 h and then harvested for cell lysis preparation. The accumulation of LC3-II over time was determined by western blot **(B)**. The expression levels of phospho-AMPK and total AMPK were detected by western blot **(C)**. Transfection of siRNA was conducted to knock down the expression of AMPK followed by treatment of JB for 48 h. Cells were collected and prepared into lysis. The expression levels of P-AMPK, LC3-I, and LC3-II were determined by western blot **(D)**.

Autophagy plays a crucial role in maintaining cellular homeostasis. It is known that autophagy occurs in response to various environmental stresses such as nutrient deficiency, growth factor deficiency, hypoxia, and various cytotoxic insults (Dikic and Elazar, 2018; Wang and Zhang, 2019). When autophagy occurs, LC3-I converts to LC3-II; meanwhile, p62 will be degraded to link ubiquitinated proteins to autophagic machinery enabling their degradation in lysosome (Bjørkøy et al., 2009). Whether JB treatment induces autophagy was studied by detecting classical landmarks of autophagy, including LC3 and p62. As shown in Figure 4A, the conversion of LC3-I to LC3-II gradually increases with the increase of JB concentration. In addition, conversion elevation rate over time was investigated. Figure 4B showed that LC3-II accumulated apparently at an early stage in only 12 h upon JB treatment, indicating that cells were sensitive to JB treatment.

The literature has shown that AMP-activated protein kinase (AMPK), a central regulator of energy homeostasis of the cell (Steinberg and Carling, 2019), is an important upstream activator regulating autophagy. Stress conditions, such as glucose starvation, oxidative stress, and hypoxia, could phosphorylate and activate AMPK (Zhao and Klionsky, 2011; Zhang and Lin, 2016). To validate whether AMPK participates in the JB-induced autophagy, the expression of phosphorylated AMPK was detected. As presented in Figure 4C, phosphorylated AMPK increased dose-dependently, suggesting that AMPK may play a role in autophagy induction. Following this finding, we then silenced the expression of AMPK by siRNA transfection to ascertain the function of AMPK in autophagy. As shown in Figure 4D, autophagy was blocked in AMPK-downregulated MCF-7. However, in AMPK-downregulated MDA-MB-231, LC3-I still converted to LC3-II, indicating that autophagy was not affected. This difference suggested there probably exists cell specificity in the mechanism of JB-induced autophagy.

### Inhibition of Jujuboside B-Induced Autophagy Enhances Apoptosis

**FIGURE 5:**
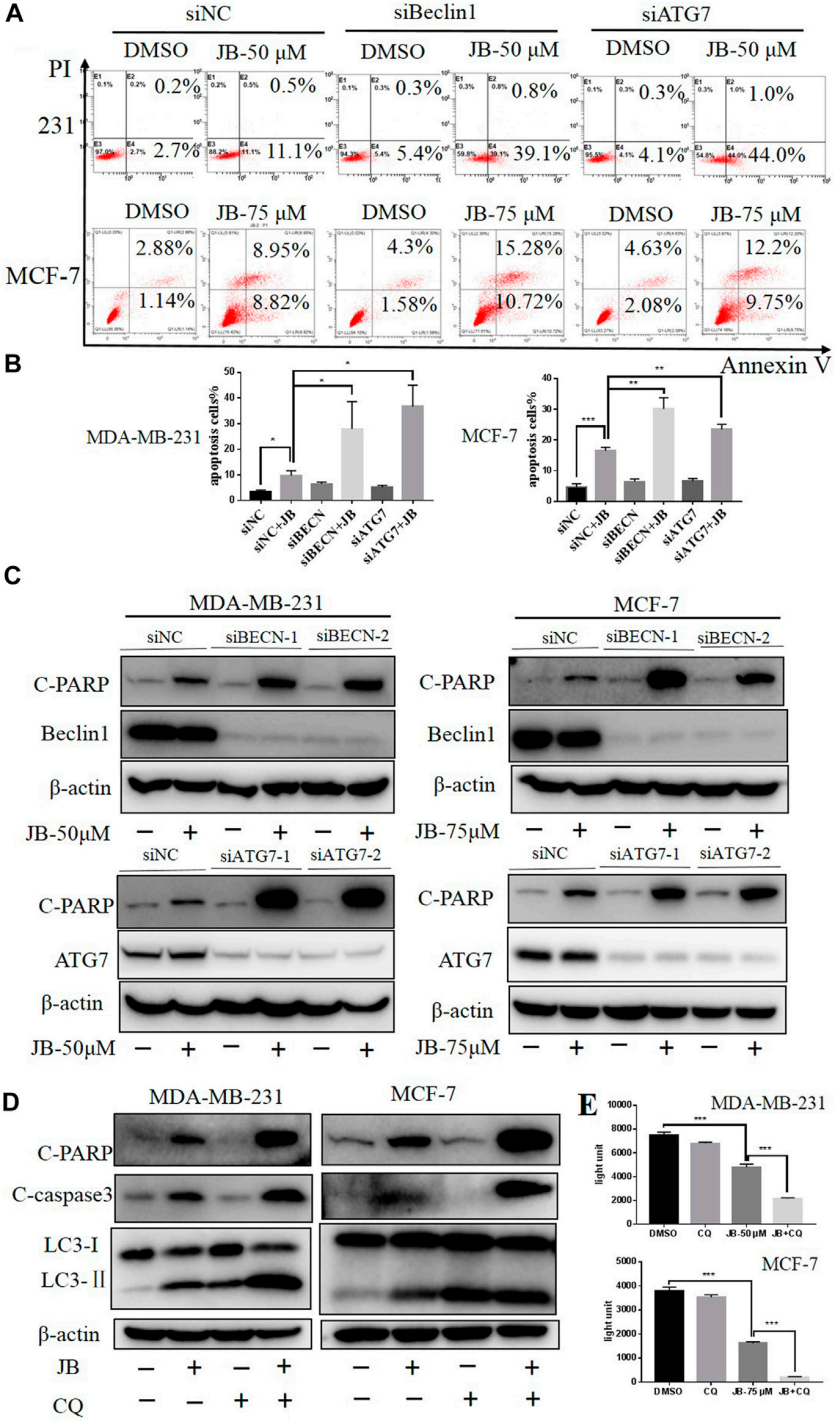
Inhibition of JB-induced autophagy enhances apoptosis. JB-induced autophagy was inhibited *via* a genetic approach using siRNA of autophagy essential gene Beclin1 and ATG7, followed by treatment of JB for 48 h. Flow cytometry was performed to detect apoptosis **(A)**, and statistical analysis of the relative apoptotic percentages were presented by mean ± SEM **(B)**. The expressions of C-PARP, ATG7, and Beclin1 were detected by western blot **(C)**. Further, CQ was used to block autophagy; then, apoptosis characterized by C-PARP and C-cas3 was determined **(D)**. Cell viability under the condition of JB and/or CQ was determined by ATPlite **(E)**. *p < 0.05; **p < 0.01; ***p < 0.001, *n* = 3.

Autophagy is a highly conserved cytoprotective process. However, it can alternatively induce autophagic cell death by over-degrading the cytoplasm (Jung et al., 2020). To clarify the function of JB-induced autophagy, we inhibited autophagy and then detected apoptosis levels. Firstly, we downregulated autophagy essential genes Beclin1 and ATG7 with two siRNA sequences, respectively. As a result, the apoptotic cell population remarkably increased, as depicted in flow cytometry analysis shown in Figures 5A,B. Consistently, the apoptosis marker cleaved PARP was significantly enhanced in the Beclin1/ATG7-downregulated cells under treatment of JB (Figure 5C). Secondly, autophagy was blocked by CQ, a typical autophagy inhibitor, and apoptosis level was detected. As shown in Figure 5D, the expression of C-PARP and C-cas3 were remarkably elevated when co-treated with JB and CQ. Moreover, a cell viability assay was performed to confirm the cell proliferation in JB- and/or CQ-treated conditions. Results indicated that inhibiting autophagy by CQ effectively promoted JB-induced proliferation inhibition (Figure 5E). These results indicated that inhibiting autophagy by both genetic and pharmacological approaches significantly enhanced apoptosis. Therefore, JB-induced autophagy acts as a pro-survival role in both MDA-MB-231 and MCF-7.

### Jujuboside B Suppresses the Tumor Growth *In Vivo*

**FIGURE 6:**
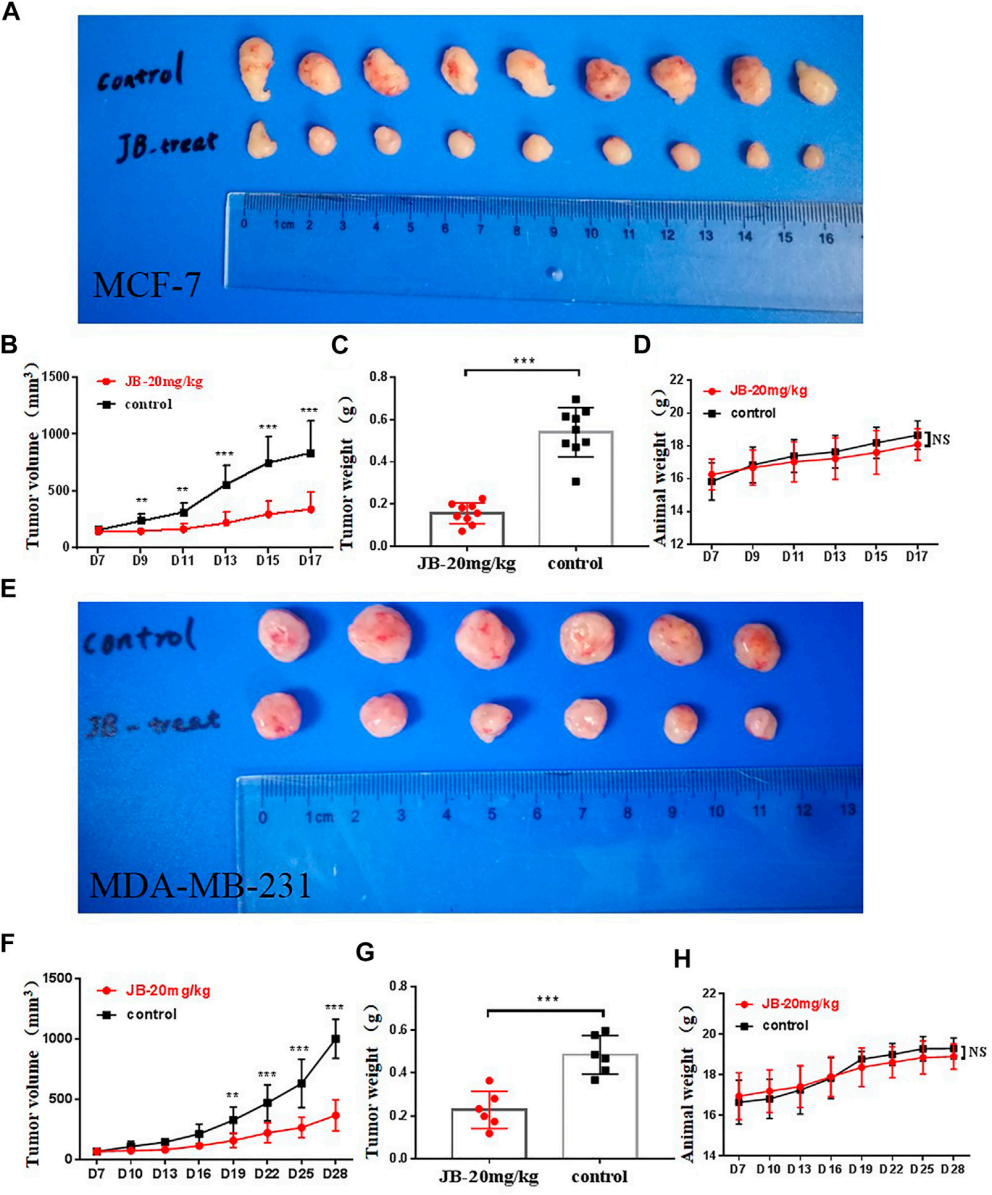
JB suppresses tumor growth *in vivo*. JB significantly inhibited the tumor growth of MCF-7 **(A,B)** and MDA-MB-231 **(E,F)**. The tumor weights of the JB-treated group were much lower than those of the control group **(C,G)**. There is no significant difference between animal weights of the JB- or vehicle-treated group **(D,H)**. The values of tumor volume and weight were analyzed and presented by mean ± SEM, *n* = 9 or 6. *p < 0.05 vs. control group; **p < 0.01 vs. control group; ***p < 0.001 vs. control group.

To further verify the anti-tumor potential of JB *in vivo*, a subcutaneous tumor model was established with MCF-7 and MDA-MB-231 cells. As shown in Figure 6, JB significantly inhibited the tumor growth of MCF-7 (Figures 6A,B) and MDA-MB-231 (Figures 6E,F). The tumor weights of the JB-treated group were much lower than those of the control group (Figures 6C,G). There is no significant difference between animal weights of the JB- and vehicle-treated group (Figures 6D,H). The results *in vivo* further validated the anti-breast cancer potential of JB. Protein samples were extracted from the tumor tissue and analyzed by western blot to detect the apoptosis and autophagy level. As shown in Supplementary Figure S1, the expression of cleaved PARP, cleaved caspase-3, and LC3-II increased in JB-treated tissue. The autophagy level in MCF-7 tissue was lower than that *in vitro*. One explanation is that the concentration of JB distributed to the tumor *in vivo* may be lower than the JB exposure of cells in the culture dish.

## Discussion

Breast cancer has become the most common cancer diagnosed among women and is the second leading cause of cancer-related death among women all over the world (DeSantis et al., 2019). Breast cancer patients generally suffer from insomnia and anxiety, resulting in a poor prognosis (Watts et al., 2014; Greenlee et al., 2017; Villar et al., 2017). Studies have shown that the patients usually get better survival and prognosis when adjuvant sedative-hypnotic Chinese medicines treatments are used (Yeung et al., 2018). However, the mechanism of this enhanced therapeutic effect has not been well studied. Zizyphi Spinosi Semen (ZSS) has a long history of sedative-hypnotic use in China. Previous studies have reported that jujuboside B (JB), a main active component of ZSS, has anti-tumor activity in colon cancer (Xu et al., 2014). These studies have led to the hypothesis that JB could attenuate breast cancer.

Indeed, our findings confirmed the anti-tumor effect of jujuboside B in breast cancer. Jujuboside B significantly inhibited the malignant proliferation of breast cancer cell lines MDA-MB-231 and MCF-7 *in vitro* and *in vivo*. It is a generally acknowledged fact that MCF-7 is low-metastatic and has little migration potential (Hu et al., 2019). MDA-MB-231 is a triple-negative breast cancer cell that is highly metastatic and prone to metastasis (Hasanpourghadi et al., 2018). Our findings also showed that JB could significantly suppress the migration of MDA-MB-231.

In this research, it was found that jujuboside B significantly induces apoptosis *in vivo* and *in vitro* characterized by elevated expression of cleaved PARP and cleaved caspase 3. The Bcl-2 family proteins participate in apoptosis by regulating the permeability of the mitochondrial outer membrane (Cory and Adams, 2002). NOXA is a pro-apoptotic subset of the Bcl-2 family proteins and it can be transactivated by the tumor suppressor p53 (Yu and Zhang, 2005; O’Prey et al., 2014). Previous studies have indicated that some compounds from traditional Chinese medicine, like resveratrol, induced apoptosis by upregulating NOXA and simultaneously downregulating Bcl-2 and Bcl-XL (Shankar et al., 2007). Therefore, we detected the expression of NOXA and found out that it mediated JB-induced apoptosis (Figure 3).

Besides, JB induced autophagy in both MDA-MB-231 and MCF-7 cells and exhibits a dose-dependent manner *in vitro*. This effect was characterized by the conversion of LC3-I to LC3-II and the decreased expression of p62. p62, as a secondary marker of autophagy, can be degraded and can link ubiquitinated proteins to the autophagic machinery to enable their degradation in the lysosomes (Bjørkøy et al., 2009; Liu et al., 2016). Autophagy is a lysosomal degradation pathway, which is essential for survival, differentiation, development, and homeostasis. Autophagy principally has an adaptive role in protecting organisms against diverse pathologies, including infections, cancer, neurodegeneration, and aging (Levine and Kroemer, 2008). In cancer, excellent works have demonstrated the dual functions of autophagy in tumor biology: autophagy activation can promote cancer cells survival (protective autophagy) or contribute to cancer cell death (autophagic cell death) (Nikoletopoulou et al., 2013; Russo and Russo, 2018). To find out whether autophagy plays a protective or accelerative role in cell death, we inhibited autophagy by two approaches and then detected the apoptosis level. Results showed that block of autophagy elevated JB-induced apoptosis, suggesting that autophagy is a protective cellular response. Previous studies have indicated that activation of AMPK promotes autophagy in multiple human cancer (Kim et al., 2011; Zhao and Klionsky, 2011). However, we found that the mechanism of JB-induced autophagy varied between these two cell lines. In MCF-7, the conversion of LC3-I to LC3-II was blocked by the knockdown of AMPK. In comparison, the accumulation of LC3-II was not affected by AMPK knockdown in MDA-MB-231. AMPK is a metabolic sensor in mammals that is activated when ATP decreases. Apart from autophagy, AMPK participates in various physiological processes, including cell growth, energy balance, lipid oxidation, glucose uptake, mitochondrial fission, and reprogramming cellular metabolism (Garcia and Shaw, 2017; Herzig and Shaw, 2018). AMPK can be extensively phosphorylated in these above physiological processes by multiple upstream signals, including LKB1, CAMKK2, and ubiquitination (Garcia and Shaw, 2017). Meanwhile, there exists crosstalk between the AMPK pathway and other cell signaling pathways like AKT, mTOR, ROS, and Ras-Raf-MEK-ERK pathway (Hardie, 2014; Zhao et al., 2017). Multiple pathways regulate autophagy, such as the mTOR pathway, hedgehog pathway, and MAPK/Erk pathway (Zeng and Ju, 2018; Wang and Zhang, 2019; Hanyu et al., 2020). Thus, the phosphorylated AMPK detected in MDA-MB-231 was not necessarily a regulator of autophagy. The underlying mechanism of JB-induced autophagy in MDA-MB-231 remains to be further explored.

In this study, we focused on whether JB has potential anti-breast cancer activity *in vitro* and *in vivo*. In order to confirm JB’s safety and toxicity, we performed an informal preliminary experiment using three doses, 20, 30, and 40 mg/kg/day. Three doses were separately given to three mice; then, we observed their liveliness and recorded body weight. As shown in Supplementary Figure S2, the mice in the 30 and 40 mg/kg/day group suffered weight loss compared with their initial body weight. During the process, mice in the 30 and 40 mg/kg/day group appeared much less vigorous than those in the 20 mg/kg/day group. In addition, the principle of medication is administrating as few drugs as possible to achieve a therapeutic effect; we try to explore the minimum dose to assure its anti-cancer effect *in vivo*. Moreover, according to the Animal Care and Use Committee, if an exploratory experiment has not been conducted before, we should use as few animals as possible to achieve the preliminary goal. We selected a single dose to preliminarily focus on whether JB has anti-breast cancer activity *in vivo* or not. It turned out to be safe and effective, as shown in Figure 6. Results showed that JB significantly inhibited the tumor growth of both MCF-7 and MDA-MB-231 *in vivo*. To better reveal the dose-effect relationship, further experiments involving multiple doses remain to be conducted in the future.

**FIGURE 7:**
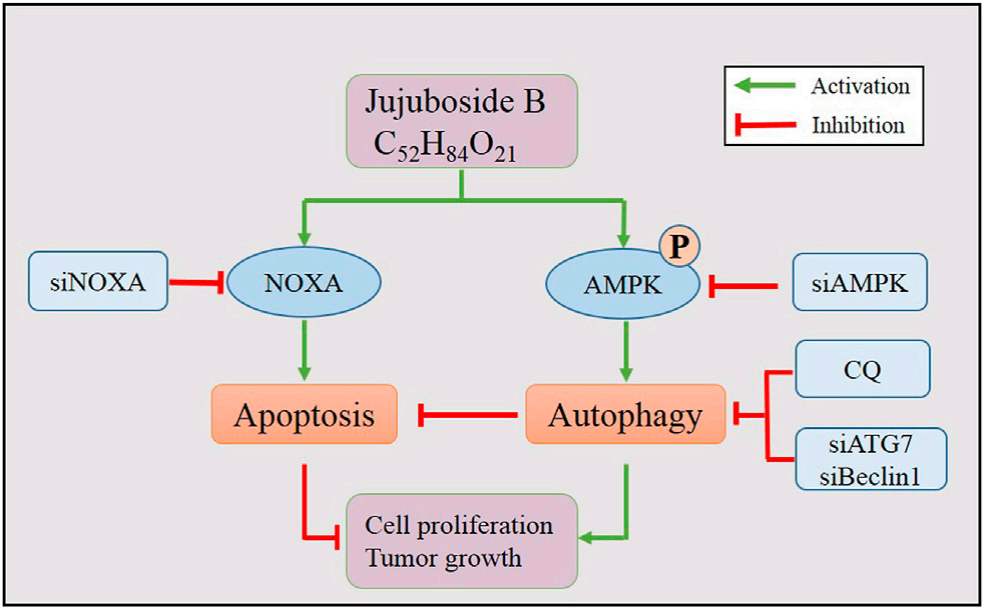
Working model of JB-induced apoptosis and autophagy.

In conclusion, our findings illustrated the anti-breast cancer efficacy of JB and elucidated its underlying mechanism (working model shown in Figure 7). This investigation may shed light on the role of JB as a potential anti-tumor agent.

## Data Availability

The raw data supporting the conclusion of this article will be made available by the authors, without undue reservation.
