# Supplementary figures and images for: Jujuboside B Inhibits the Proliferation of Breast Cancer Cell Lines by Inducing Apoptosis and Autophagy
Source: Front Pharmacol. 2021 Sep 24;12:668887. doi: 10.3389/fphar.2021.668887 (PMC8497973; doi:10.3389/fphar.2021.668887)

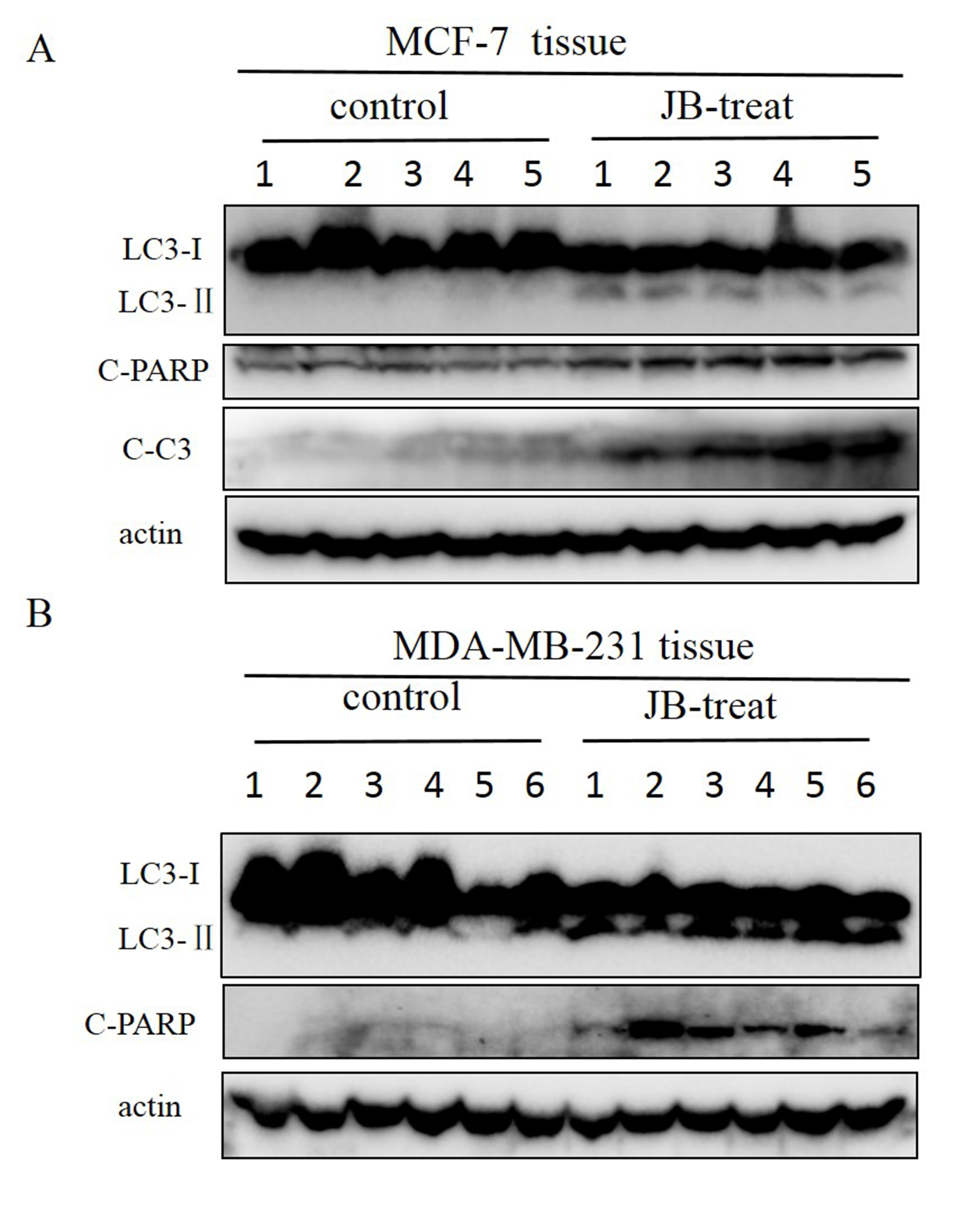

Supplement: Supplementary file 1 [file Image1.jpeg]

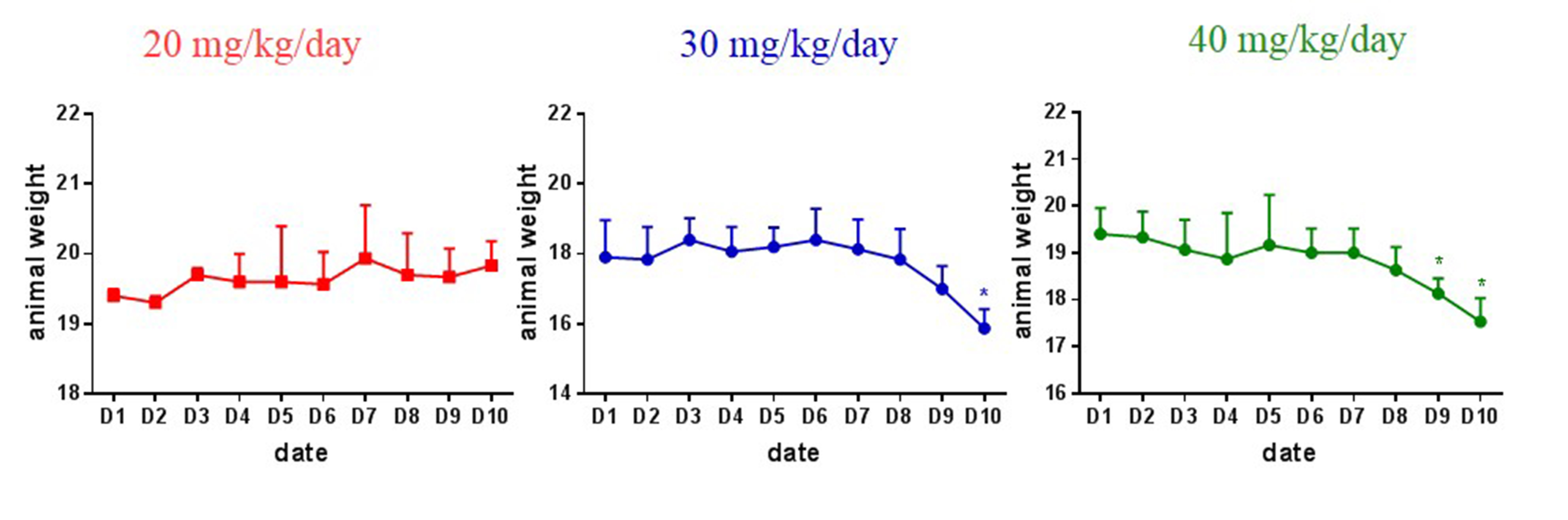

Supplement: Supplementary file 2 [file Image2.jpeg]
